# Factors associated with health-related quality of life in patients undergoing percutaneous coronary intervention: Thai PCI registry

**DOI:** 10.3389/fcvm.2023.1260993

**Published:** 2023-11-08

**Authors:** Sukanya Siriyotha, Oraluck Pattanaprateep, Suphot Srimahachota, Nakarin Sansanayudh, Ammarin Thakkinstian, Thosaphol Limpijankit

**Affiliations:** ^1^ Department of Clinical Epidemiology and Biostatistics, Faculty of Medicine Ramathibodi Hospital, Mahidol University, Bangkok, Thailand; ^2^ Cardiac Center and Division of Cardiovascular Medicine, King Chulalongkorn Memorial Hospital and Chulalongkorn University, Bangkok, Thailand; ^3^ Cardiology Unit, Department of Internal Medicine, Pharmongkutklao Hospital, Bangkok, Thailand; ^4^ Division of Cardiology, Department of Medicine, Faculty of Medicine Ramathibodi Hospital, Mahidol University, Bangkok, Thailand

**Keywords:** health-related quality of life (HRQoL), utility scores, EQ-5D-5l, percutaneous coronary intervention (PCI), coronary artery disease (CAD)

## Abstract

**Background:**

Percutaneous coronary intervention (PCI) has been shown to improve health-related quality of life (HRQoL) in patients with coronary artery disease (CAD). The objectives of this study were to assess the changes in HRQoL and factors influencing these changes in CAD patients after undergoing PCI.

**Methods:**

Data from a nationwide PCI registry across 39 hospitals in Thailand were collected in 2018–2019, including baseline characteristics, comorbid diseases, angiographic CAD severity, procedural details, and type of health insurance. HRQoL, as measured by utility scores, was determined in all patients using the Thai version of EQ-5D-5l at admission, discharge, and 6 and 12 months after discharge. The effects of time after PCI procedure and various factors on mean utility scores were assessed using a mixed-effect linear regression model.

**Results:**

A total of 19,701 patients were included in the analysis; they had a mean age of 64.2 ± 11.7 years and were predominantly (69.1%) male. Following PCI, the mean utility scores increased from 66.6 ± 19.6 at admission to 81.9 ± 13.8 at discharge, and remained stable at 6 and 12 months (86.1 ± 12.3 and 88.0 ± 11.7, respectively). After adjusting for potential confounding variables, several factors were found to be independently associated with improved HRQoL, including angiographic success, male gender, overweight status, dyslipidemia, and radial access. Six other factors were associated with less improved HRQoLs, including cardiogenic shock/IABP support, old age, CKD, clinical presentation (STEMI and NSTEMI), prior cerebrovascular disease, and heart failure. There were no associations of CAD severity and procedural details with HRQoL. No differences were found related to type of health insurance, except that patients who were uninsured or self-pay tended to have less improvement in HRQoL.

**Conclusion:**

HRQoL improved significantly after PCI in these subjects, as observed through 1 year of follow-up. Identifying the factors influencing these improvements may assist clinicians in tailoring patient interventions to optimise quality of life after PCI.

## Introduction

Coronary artery disease (CAD) is a common non-communicable disease which causes angina (chest pain) and shortness of breath, limits life-style activities, and decreases longevity (1). These symptoms are due to the narrowing or blocking of blood vessels by plaque of oxidised fatty cholesterol deposited on the coronary vessel walls which impede the blood flow to heart muscle (2). Percutaneous coronary intervention (PCI) is a common medical procedure used to treat CAD, involving the dilation of blocked vessels with balloon catheters and the placement of stents to restore blood flow. PCI can alleviate angina and enhance exercise tolerance in these patients, while reducing the risk of major adverse cardiovascular events (MACEs), such as myocardial infarction (MI) and death (3, 4).

Based on the 2011 American College of Cardiology Foundation (ACCF)/American Heart association (AHA)/Society for Cardiovascular Angiography and Interventions (SCAI) Guidelines for PCI, the success of a PCI procedure is defined by three components: angiographic findings, procedural events, and clinical outcomes (5). However, CAD patients usually experience physical, psychosocial, and emotional symptoms which may negatively affect their health-related quality of life (HRQoL) (6–9). The term HRQoL is defined by the World Health Organization (WHO) as, “an individual's perception of their position in life, considering the culture, value systems, goals, expectations, standards, and concerns within their environment” (10).

There are many reports that patient HRQoL changed after undergoing PCI (11–15). Such changes are complex and influenced by multiple factors, including physical health, psychological state, personal beliefs, social relationships, and environmental factors. Utility tools, such as disease-specific questionnaires (e.g., Seattle Angina Questionnaire) and general ones [e.g., EuroQoL-5 Dimensions 5-Level (EQ-5D-5l) and EuroQoL Visual Analogue Scale (EQ-VAS)] (16–18) have been used to estimate HRQoL at a specific point in time. Some suggest that a poorer HRQoL or lower utility score is associated with increased mortality and MACEs, even in patients who have undergone successful PCI (19).

In Thailand, there is little data focused on the HRQoL of CAD patients (20). Furthermore, large-scale studies of HRQoL changes and the factors which influence these changes in CAD patients who undergo PCI are very limited. Therefore, we conducted this study to assess the HRQoL changes and influencing factors in CAD patients during the first 12 months following PCI using a Thai PCI registry.

## Materials and methods

This study utilised data from a nationwide prospective multicenter Thai PCI Registry, initiated in 2018 by the Cardiac Intervention Association of Thailand (21). Briefly, it included data from 39 hospitals (university, government and private) located in five regions of the country which voluntarily participated. All adult patients enrolled in this study were aged 18 years or older, and underwent PCI between May 1, 2018, and April 2, 2019, as well as between June 21 and August 1, 2019. The study was approved by the Central Research Ethics Committee (COA-CREC # 006/2018) and the Ethics Committee of the Faculty of Medicine, Ramathibodi Hospital, Mahidol University (COA-MURA2022/205). All patients provided written informed consent.

### Data collection

Clinical and angiographic characteristics, along with procedural data, were retrieved from the registry's main electronic databases. Patient data for analysis included: age, gender, health insurance (universal coverage, government service/state enterprise, social security service, uninsured or self-pay), body-mass index (BMI), presence of cardiovascular risk factors [diabetes mellitus (DM), hypertension, dyslipidemia, chronic kidney disease (CKD, defined as eGFR < 60 ml/min/1.73 m^2^), and smoking] as well as history of related underlying diseases (cerebrovascular disease, MI, heart failure and previous PCI/CABG). Clinical and angiographic data collected included: clinical presentation [ST-elevation myocardial infarction (STEMI), non-ST-elevation myocardial infarction (NSTEMI)/unstable angina (UA) and stable CAD], number of diseased vessels, left ventricular ejection fraction (LVEF), presence of cardiogenic shock or intra-aortic balloon pump (IABP) insertion, radial access, lesion severity assessment (intravascular ultrasound study, optical coherence tomography or fractional flow reserve wire), plaque modification device used (rotational atherectomy, cutting/scoring balloon or laser atherectomy), numbers of chronic occlusion lesions, lesions treated (1, ≥1), treated vessels (1, 2, ≥3), and stents used (1, 2, ≥3). Additionally, intra- and post-procedural events [angiographic success (residual stenosis <20% with stent treatment, or <50% with balloon angioplasty alone) and procedural complications] were noted. Procedural complications were also recorded, including death, MI, stroke, cardiogenic shock, heart failure, new requirement of dialysis, bleeding (within 72 h or requiring transfusion), endotracheal intubation, cardioversion/defibrillation, and in-hospital CABG.

### Outcomes of interest

The HRQoL was measured using the Thai EQ-5D-5l (22) at admission, and discharge, 6 and 12 months after PCI procedure. The EQ-5D-5l is a self-reported description of the patient's current health in five dimensions which includes mobility, self-care (washing or dressing oneself), usual activities (such as work, study, housework, family or leisure activities), pain/discomfort and anxiety/depression. For patients who presented with unconsciousness or unstable hemodynamics, the HRQoL was assessed later when they were stable and able to provide information.

The EQ-5D-5l questionnaire consisted of five Likert scales ranging from “no problems” to “unable/extreme problems”. The score profile was then converted to a utility score using Thai coefficients multiplied by 100. The utility score ranged from −28.30 to 100.00, of which <0, 0, and 100 represented worse than death, death, and perfect HRQoL, respectively.

### Statistical analysis

Patient characteristics were summarized using mean ± SD for continuous data and frequency (with percentage) for categorical data. A univariate mixed-effect regression model was performed by regressing repeatedly measured utility scores on time (admission, discharge, 6 months, and 12 months after discharge from PCI procedure) and the other 26 covariates. These are listed in Table 1 [i.e., 3 demographic (age, gender and BMI), health insurance and clinical presentation, 5 cardiovascular risk factors (i.e., DM, hypertension, dyslipidemia, CKD and smoking), 5 underlying cardiovascular diseases (i.e., cerebrovascular disease, MI, stable CAD, heart failure and previous PCI/CABG), 11 diseased coronary vessels (disease severity, LVEF, number of diseased vessels, cardiogenic shock, radial access, lesion severity, number of lesions treated, number of stents used, CTO lesion, angiographic success and procedure complications)]. Covariables in a univariate analysis whose *p*-values were less than 0.1 were simultaneously included in a multivariate, mixed-effect, linear regression model and those remaining significant were incorporated into the final equation. Potential interactions between covariates were explored. Adjusted coefficients of association, along with 95% confidence intervals (CI), were then estimated. Moreover, serial changes of HRQoL within-group, of each factor, were also calculated. All analyses were performed based on complete-case data using STATA 18.0 (Stata Corp., TX, USA). A *p*-value of less than 0.05 was considered statistically significant.

**Table 1:** Baseline characteristics of patients and PCI outcomes.

Characteristics	*N* = 19,701
Age groups, years, *n* (%)	
<45	1,070 (5.4)
45–64	9,216 (46.8)
65–79	7,588 (38.5)
≥80	1,827 (9.3)
Male gender, *n* (%)	13,618 (69.1)
BMI ≥ 23 kg/m^2^, *n* (%)	11,882 (60.3)
DM, *n* (%)	8,703 (44.2)
Hypertension, *n* (%)	13,286 (67.4)
Dyslipidemia, *n* (%)	12,857 (65.3)
Cerebrovascular disease, *n* (%)	1,117 (5.7)
CKD, *n* (%)	
With dialysis	689 (3.5)
Without dialysis	5,702 (28.9)
No	13,310 (67.6)
Current smoker	4,608 (23.4)
History of MI, *n* (%)	4,575 (23.2)
History of heart failure, *n* (%)	2,686 (13.6)
Previous PCI, *n* (%)	5,853 (29.7)
Previous CABG	309 (1.6)
Types of health insurance, *n* (%)	
Universal coverage	12,534 (63.6)
Government service/state enterprise	5,237 (26.6)
Social security service	1,329 (6.7)
Uninsured or self-pay	601 (3.1)
CAD presentation, *n* (%)	
STEMI	5,479 (27.8)
NSTEMI/unstable angina	5,976 (30.3)
Stable CAD	8,246 (41.9)
Number of diseased vessels, *n* (%)	
Single vessel	5,202 (26.4)
Double vessel	5,675 (28.8)
Triple vessel/LM	8,824 (44.8)
LVEF < 40%, *n* (%)	2,806 (22.3)
Cardiogenic shock/IABP, *n* (%)	1,714 (8.7)
Radial access, *n* (%)	8,681 (44.1)
Lesion severity assessment, *n* (%)	3,140 (15.9)
Plaque modification devices, *n* (%)	1,022 (5.2)
CTO lesions, *n* (%)	1,969 (10.0)
Number of lesions treated, *n* (%)	
1	15,528 (78.8)
>1	4,173 (21.2)
Number of treated vessels, *n* (%)	
1	17,056 (86.6)
2	2,444 (12.4)
≥3	201 (1.0)
Number of stents used, *n* (%)	
1	10,052 (55.3)
2	5,620 (30.9)
≥3	2,515 (13.8)
Angiographic success, *n* (%)	18,780 (95.3)
Procedural complications, *n* (%)	1,021 (5.2)

## Results

### Baseline characteristics and procedural details

A total of 19,701 patients were included in the analysis (Table 1), of which 17,432 patients were followed up at 1 year. Sixty-nine percent of patients were male. The majority (85.3%) were aged 45–79 years, while only 5.4% were younger than 45 years old, and 9.3% were 80 years or older. Approximately 60% of patients were classified as overweight (BMI ≥ 23 kg/m^2^). Common risk factors included hypertension (67.4%), dyslipidemia (65.3%), DM (44.2%), CKD with (3.5%) or without dialysis (28.9%), and current smoking (23.4%). A prior history of MI, heart failure and previous PCI were present in 23.3%, 13.6% and 29.7%, respectively. The majority of patients were insured through universal health coverage (63.6%), followed by government service/state enterprise (26.6%), and social security service (6.7%). Approximately 58.1% of patients exhibited acute coronary syndrome (ACS; either STEMI or NSTEMI/UA), as the primary clinical presentation, while 41.9% presented with stable CAD. Nearly half of the patients had triple vessel or left main disease. Twenty-two percent of patients had impaired LV systolic function (LVEF < 40%), and 8.7% presented with cardiogenic shock and/or required IABP support. The angiography was successful in 95.3% of subjects, while a procedural complication occurred in 5.2%.

### Health-related quality of life changes after PCI

Of the 19,701 patients, only 17,432 completed the questionnaires due to in-hospital/follow-up death (10.5%) and loss to follow-up (1.1%). After undergoing PCI, patients' mean utility scores increased, rising from 66.6 ± 19.6 at admission to 81.9 ± 13.8 at discharge. These scores increased further, reaching 86.1 ± 12.3 and 88.0 ± 11.7 at 6 and 12 months, respectively. The distributions of EQ-5D-5l and VAS scores for the study cohort are presented in Supplementary Table S1. Individual HRQoL domains improved after PCI procedure, particularly the level of pain or discomfort (moderate to extreme levels), which decreased from 33.2% at admission to 7.3%, 4.2%, and 3.2% at discharge, 6 and 12 months, respectively. Similarly, anxiety or depression decreased from 26.9% to 5.8%, 2.6% and 2.1% at these respective time points. Components related to functional class, such as mobility, self-care and usual activities, also improved following PCI both by discharge and during the 12-month follow-up period.

### Factors associated with HRQoL after PCI

The factors associated with HRQoL were assessed and are summarized in Table 2. Overall, HRQoL increased over time and was significantly improved about 10 points after performing PCI, i.e., from 73.5 to 85.6, 86.3, and 85.6 at discharge, 6- and 12-months post-PCI, respectively. A univariate mixed-effect linear regression analysis revealed significant increases in HRQoL at these three time points compared to pre-procedural levels. The coefficients of association (95% CI) for these time points were 12.1 (11.8, 12.5), 12.8 (12.4, 13.2), and 12.1 (11.7, 12.5), respectively (Table 2). Similar trends were observed in within-group of each factor. For instance, elderly patients (particularly octogenarians), females, those with a lower BMIs (<23 kg/m^2^), current smokers, and those with comorbid diseases (such as DM, CKD, prior cerebrovascular disease, heart failure or previous CABG) had lower baseline HRQoLs than after the PCI procedure. Similarly, patients who presented with ACS and LVEF <40%, or experienced cardiogenic shock or needed IABP support, also had lower HRQoLs. In addition, a univariate mixed-effect linear regression analysis also revealed significant differences of HRQoL between-groups of factors. For instance, the coefficients of HRQoLs were 2.0, 8.4 and 22.1 lower for the groups aged 45–64, 65–79 and ≥80 years when compared to the <45-year-old age group.

**Table 2:** Predictors associated with HRQoL scores: a univariate analysis.

Factors	Admit	Discharge	At 6 months	At 12 months	Overall	Coef. (95% CI)	*p*-value
	Mean ± SD	Mean ± SD	Mean ± SD	Mean ± SD	Mean ± SE		
Time							
Follow up 12 months					85.6 ± 0.2	12.1 (11.7, 12.5)	<0.001
Follow up 6 months					86.3 ± 0.2	12.8 (12.4, 13.2)	<0.001
Discharge					85.6 ± 0.2	12.1 (11.8, 12.5)	<0.001
Admission					73.5 ± 0.2	0	
Age groups, year							
≥80	63.9 ± 34.4	69.0 ± 37.9	66.4 ± 40.2	74.1 ± 30.7	68.5 ± 0.5	−22.1 (−23.7, −20.5)	<0.001
65–79	74.5 ± 29.4	83.5 ± 29.1	82.7 ± 31.2	84.4 ± 23.3	81.4 ± 0.3	−8.4 (−9.8, −7.0)	<0.001
45–64	78.0 ± 28.3	91.1 ± 22.0	91.0 ± 23.5	89.2 ± 18.4	87.5 ± 0.2	−2.0 (−3.4, −0.6)	0.004
<45	77.7 ± 29.2	94.2 ± 18.2	93.6 ± 20.8	91.1 ± 16.0	89.4 ± 0.7	0	
Gender							
Male	77.1 ± 28.9	88.21 ± 25.7	87.7 ± 27.6	87.8 ± 20.7	85.3 ± 0.2	6.1 (5.5, 6.8)	<0.001
Female	71.6 ± 30.8	82.12 ± 30.2	81.1 ± 32.5	82.4 ± 24.6	79.4 ± 0.3	0	
BMI ≥ 23 kg/m^2^							
Yes	76.4 ± 28.6	88.7 ± 24.4	88.1 ± 26.4	87.3 ± 20.4	85.3 ± 0.2	4.7 (4.1, 5.4)	<0.001
No	73.8 ± 31.0	82.8 ± 30.9	81.9 ± 32.9	84.3 ± 24.2	80.9 ± 0.3	0	
Types of health insurance							
Government service/state enterprise	76.9 ± 28.3	86.1 ± 26.8	85.3 ± 28.6	85.2 ± 22.4	83.6 ± 0.3	0.6 (−0.1, 1.3)	0.099
Social security service	78.3 ± 28.5	90.9 ± 22.2	91.1 ± 23.8	89.2 ± 18.7	87.5 ± 0.6	4.7 (3.4, 5.9)	<0.001
Uninsured or self-pay	75.3 ± 27.3	87.1 ± 26.1	86.5 ± 27.7	84.7 ± 22.2	83.4 ± 0.9	0.8 (−1.1, 2.6)	0.405
Universal coverage	74.4 ± 30.3	85.9 ± 28.1	85.2 ± 30.0	86.0 ± 22.2	83.1 ± 0.2	0	
Clinical presentation							
STEMI	61.6 ± 37.0	81.4 ± 33.6	81.3 ± 34.8	81.5 ± 28.2	77.3 ± 0.3	−12.2 (−13.0, −11.5)	<0.001
NSTEMI/unstable angina	74.5 ± 29.0	85.5 ± 27.8	84.8 ± 29.9	85.4 ± 22.1	82.6 ± 0.3	−5.9 (−6.6, −5.2)	<0.001
Stable CAD	83.0 ± 22.1	90.2 ± 21.1	89.2 ± 24.0	89.6 ± 16.1	88.0 ± 0.2	0	
DM							
Yes	73.6 ± 30.1	83.3 ± 30.1	82.3 ± 32.3	83.8 ± 24.3	80.9 ± 0.2	−5.0 (−5.6, −4.4)	<0.001
No	76.8 ± 29.2	88.7 ± 24.7	88.4 ± 26.4	87.9 ± 20.0	85.6 ± 0.2	0	
Hypertension							
Yes	76.2 ± 28.3	85.3 ± 27.8	84.4 ± 30.1	85.4 ± 22.3	82.9 ± 0.2	−1.7 (−2.4, −1.0)	<0.001
No	73.6 ± 32.2	88.4 ± 26.2	88.3 ± 27.6	87.6 ± 21.5	84.7 ± 0.3	0	
Dyslipidemia							
Yes	77.7 ± 27.2	87.0 ± 26.0	86.3 ± 28.1	86.7 ± 20.7	84.5 ± 0.2	3.3 (2.7, 4.0)	<0.001
No	70.8 ± 33.4	85.2 ± 29.7	84.5 ± 31.6	85.0 ± 24.4	81.7 ± 0.3	0	
Cerebrovascular disease							
Yes	66.2 ± 33.5	74.7 ± 35.7	74.1 ± 36.7	76.5 ± 29.9	73.1 ± 0.7	4.2 (3.1, 5.3)	<0.001
No	75.9 ± 29.3	87.0 ± 26.6	86.4 ± 28.7	86.7 ± 21.4	84.1 ± 0.2	0	
CKD							
With dialysis	65.5 ± 33.2	67.7 ± 39.5	63.3 ± 41.6	74.8 ± 29.6	67.8 ± 0.8	−19.8 (−21.4, −18.1)	<0.001
Without dialysis	71.5 ± 31.7	78.5 ± 34.1	76.8 ± 36.4	80.3 ± 27.9	76.9 ± 0.3	−11.0 (−11.7, −10.4)	<0.001
No	77.4 ± 28.2	90.6 ± 21.6	90.6 ± 23.0	89.1 ± 17.8	87.1 ± 0.2	0	
Current smoker							
Yes	70.6 ± 33.4	87.7 ± 27.2	87.6 ± 28.8	86.4 ± 22.8	83.4 ± 0.2	−12.0 (−13.3, −10.6)	<0.001
No	76.8 ± 28.3	85.9 ± 27.4	85.1 ± 29.5	86.0 ± 21.8	83.6 ± 0.3	0	
History of MI							
Yes	78.7 ± 26.5	87.4 ± 25.3	86.7 ± 27.6	87.0 ± 20.2	85.0 ± 0.3	2.3 (1.5, 3.0)	<0.001
No	74.3 ± 30.5	86.0 ± 27.9	85.4 ± 29.8	85.8 ± 22.6	83.1 ± 0.2	0	
History of heart failure							
Yes	71.3 ± 31.3	76.4 ± 35.6	74.4 ± 38.0	80.2 ± 27.7	75.7 ± 0.2	−9.7 (−10.6, −8.8)	<0.001
No	76.0 ± 29.3	87.9 ± 25.5	87.4 ± 27.3	87.0 ± 20.9	84.7 ± 0.4	0	
Previous PCI							
Yes	82.4 ± 23.1	88.6 ± 23.6	87.5 ± 26.3	88.6 ± 18.2	86.8 ± 0.3	5.3 (4.6, 6.0)	<0.001
No	72.1 ± 31.6	85.4 ± 28.7	84.9 ± 30.5	85.0 ± 23.5	82.1 ± 0.2	0	
Previous CABG							
Yes	71.3 ± 29.6	81.6 ± 28.6	78.8 ± 32.2	81.5 ± 24.8	78.4 ± 1.3	−5.0 (−7.5, −2.5)	<0.001
No	75.5 ± 29.6	86.4 ± 27.3	85.8 ± 29.3	86.2 ± 22.0	83.6 ± 0.2	0	
Number of diseased vessels							
Triple vessel/LM	77.2 ± 28.2	84.6 ± 28.7	83.6 ± 31.1	85.2 ± 23.1	82.8 ± 0.2	−1.1 (−1.9, −0.4)	0.004
Double vessel	76.4 ± 29.1	87.3 ± 26.7	86.9 ± 28.2	86.9 ± 21.3	84.5 ± 0.3	0.8 (−0.1, 1.6)	0.074
Single vessel	71.1 ± 32.0	88.1 ± 25.4	87.9 ± 27.2	86.7 ± 21.0	83.8 ± 0.3	0	
LVEF < 40%							
Yes	70.9 ± 32.6	77.5 ± 35.4	76.1 ± 37.4	80.5 ± 27.8	76.3 ± 0.4	−9.7 (−10.6, −8.8)	<0.001
No	76.2 ± 27.8	88.7 ± 22.8	88.4 ± 25.1	87.3 ± 18.9	85.2 ± 0.2	0	
Cardiogenic shock/IABP							
Yes	45.4 ± 42.2	60.7 ± 44.6	60.7 ± 45.7	61.4 ± 40.3	58.4 ± 0.5	−30.0 (−31.1, −29.0)	<0.001
No	77.1 ± 27.8	88.8 ± 23.7	88.1 ± 26.0	88.3 ± 18.1	85.6 ± 0.2	0	
Radial access							
Yes	76.2 ± 28.8	89.9 ± 22.5	89.5 ± 24.5	89.5 ± 16.9	86.5 ± 0.2	5.9 (5.3, 6.6)	<0.001
No	74.8 ± 30.2	83.5 ± 30.3	82.6 ± 32.3	83.4 ± 25.1	81.2 ± 0.2	0	
Lesion severity assessment							
Yes	75.8 ± 29.4	87.6 ± 24.7	87.5 ± 26.5	86.8 ± 20.0	84.5 ± 0.4	1.4 (0.6, 2.3)	0.001
No	75.3 ± 29.7	86.1 ± 27.8	85.3 ± 29.8	86.0 ± 22.5	83.3 ± 0.2	0	
Plaque modification devices							
Yes	76.9 ± 26.0	82.6 ± 28.6	81.6 ± 31.7	83.1 ± 23.0	81.1 ± 0.7	−2.5 (−3.9, −1.1)	0.001
No	75.3 ± 29.8	86.6 ± 27.3	85.9 ± 29.2	86.3 ± 22.0	83.7 ± 0.2	0	
Number of lesions treated							
>1	78.7 ± 28.0	85.9 ± 23.1	85.8 ± 28.2	84.8 ± 30.3	83.9 ± 0.4	0.5 (−0.3, 1.2)	0.241
1	74.5 ± 30.2	86.1 ± 21.8	86.5 ± 27.1	85.9 ± 29.1	83.4 ± 0.2	0	
Number of treated vessels							
≥3	81.2 ± 26.3	84.5 ± 28.1	81.8 ± 31.9	85.4 ± 24.1	83.3 ± 1.6	−0.5 (−3.6, 2.6)	0.747
2	79.6 ± 26.1	85.4 ± 28.7	84.5 ± 30.5	85.1 ± 23.7	83.8 ± 0.5	0.1 (−0.9, 1.0)	0.903
1	74.7 ± 30.1	86.5 ± 27.1	85.9 ± 29.1	86.2 ± 21.8	83.5 ± 0.2	0	
Number of stents used							
≥3	79.3 ± 26.7	85.6 ± 28.5	84.2 ± 31.2	86.4 ± 22.2	83.9 ± 0.4	−0.0 (−1.0, 0.9)	0.994
2	77.3 ± 28.0	87.2 ± 26.4	86.2 ± 28.7	87.2 ± 20.5	84.6 ± 0.3	0.8 (0.1, 1.5)	0.035
1	73.6 ± 30.9	87.4 ± 25.8	87.3 ± 27.4	86.6 ± 21.2	83.9 ± 0.2	0	
CTO lesions							
Yes	78.8 ± 26.6	87.4 ± 25.4	86.6 ± 28.3	86.7 ± 20.3	84.9 ± 0.5	1.9 (0.9, 3.0)	<0.001
No	75.0 ± 29.9	86.2 ± 27.6	85.6 ± 29.4	86.0 ± 22.3	83.4 ± 0.2	0	
Angiographic success							
Yes	75.4 ± 29.6	86.8 ± 26.8	86.2 ± 28.8	86.5 ± 21.6	83.9 ± 0.2	7.8 (6.3, 9.3)	<0.001
No	75.9 ± 29.0	77.1 ± 35.3	75.8 ± 38.0	78.3 ± 29.7	76.8 ± 0.7	0	
Procedural complications							
Yes	73.5 ± 30.6	77.2 ± 37.1	76.7 ± 38.5	79.0 ± 31.9	76.7 ± 0.7	−8.2 (−9.6, −6.8)	<0.001
No	75.5 ± 29.5	86.8 ± 26.6	86.2 ± 28.7	86.5 ± 21.4	83.9 ± 0.2	0	

Abbreviation as in Table 1.

**Figure 1:**
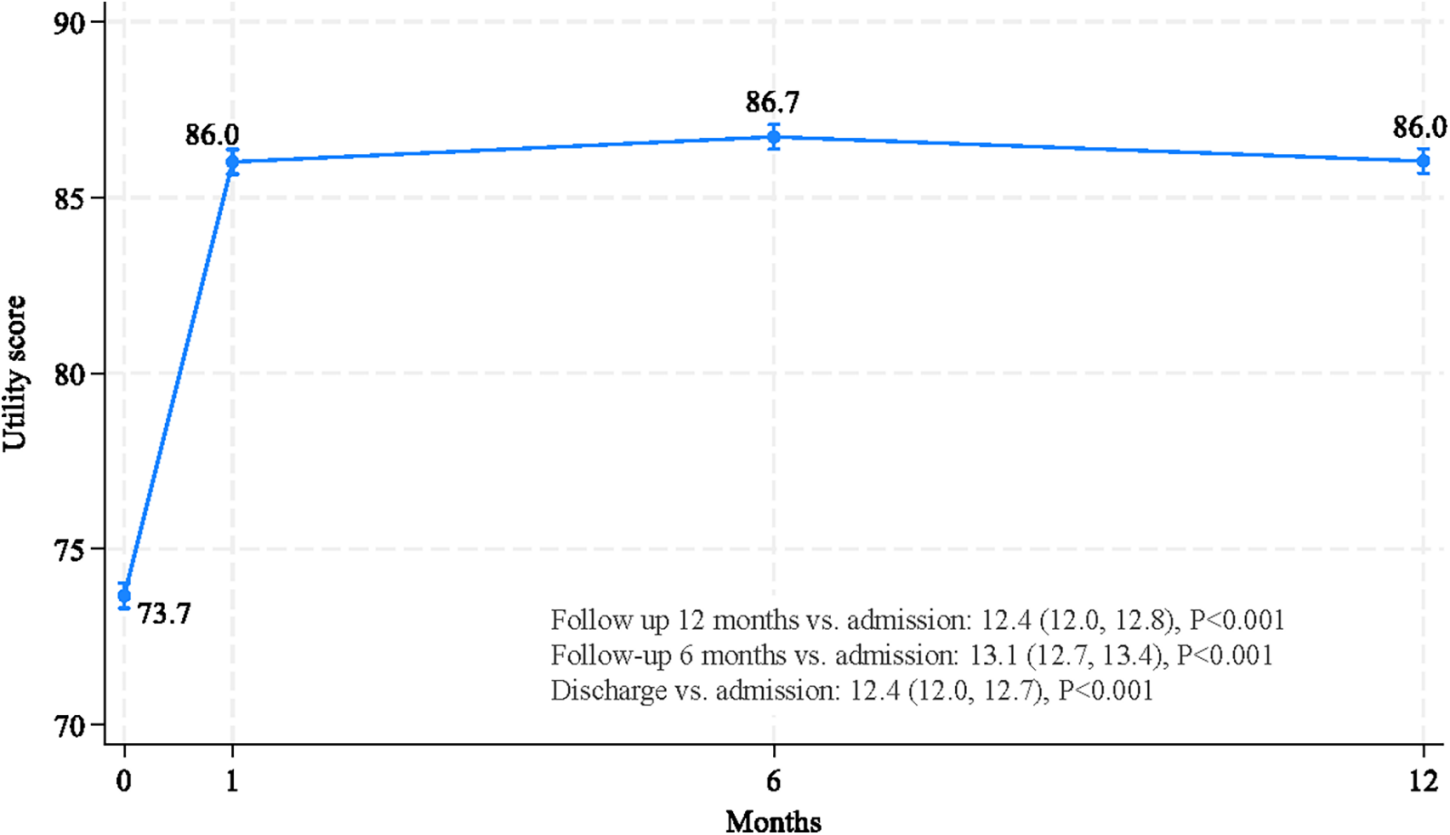
Improvements in HRQoL, as measured by utility scores, at discharge, and 6 and 12 months after PCI.

A multivariate, mixed-effect regression analysis was conducted, demonstrating that time after PCI and 16 factors were independently associated with HRQoL (Table 3). After adjusting for other factors, HRQoLs at discharge, 6 months, and 12 months following PCI significantly improved and were significantly higher than before the procedure, with coefficients (95% CI) of 12.4 (12.0, 12.7), 13.1 (12.7, 13.4) and 12.4 (12.0, 12.8), respectively (see Figure 1).

**Table 3:** Factors associated with HRQoL: a multivariate analysis.

Predictors	Coef. (95% CI)	*p*-value
Time		
Follow up at 12 months	12.4 (12.0, 12.8)	<0.001
Follow up at 6 months	13.1 (12.7, 13.4)	<0.001
Discharge	12.4 (12.0, 12.7)	<0.001
Admission	0	
Age groups, year		
≥80	−15.6 (−17.2, −14.1)	<0.001
65–79	−5.5 (−6.8, −4.2)	<0.001
45–64	−1.5 (−2.7, −0.2)	0.019
<45	0	
Gender		
Male	3.4 (2.8, 4.0)	<0.001
Female	0	
BMI ≥ 23 kg/m^2^		
Yes	1.4 (0.8, 2.0)	<0.001
No	0	
Clinical presentation		
STEMI	−9.2 (−10.0, −8.5)	<0.001
NSTEMI/unstable angina	−4.3 (−4.9, −3.6)	<0.001
Stable CAD	0	
DM		
Yes	−2.1 (−2.7, −1.5)	<0.001
No	0	
Hypertension		
Yes	−1.0 (−1.6, −0.3)	0.003
No	0	
Dyslipidemia		
Yes	1.9 (1.3, 2.5)	<0.001
No	0	
Cerebrovascular disease		
Yes	−8.7 (−9.9, −7.5)	<0.001
No	0	
CKD		
With dialysis	−15.4 (−16.9, −13.9)	<0.001
Without dialysis	−4.5 (−5.1, −3.9)	<0.001
No	0	
History of heart failure		
Yes	−5.5 (−6.3, −4.7)	<0.001
No	0	
Previous CABG		
Yes	−3.0 (−5.2, −0.9)	0.006
No	0	
Types of health insurance		
Government service/state enterprise	0.1 (−0.6, 0.7)	0.874
Social security service	−0.4 (−1.5, 0.7)	0.493
Uninsured or self-pay	−2.4 (−4.0, −0.8)	0.003
Universal coverage	0	
Cardiogenic shock/IABP		
Yes	−22.8 (−23.8, −21.8)	<0.001
No	0	
Radial access		
Yes	2.9 (2.3, 3.4)	<0.001
No	0	
Angiographic success		
Yes	6.3 (5.1, 7.6)	<0.001
No	0	
Procedural complications		
Yes	−2.1 (−3.3, −0.9)	0.001
No	0	

Abbreviation as in Table 1.

**Figure 2:**
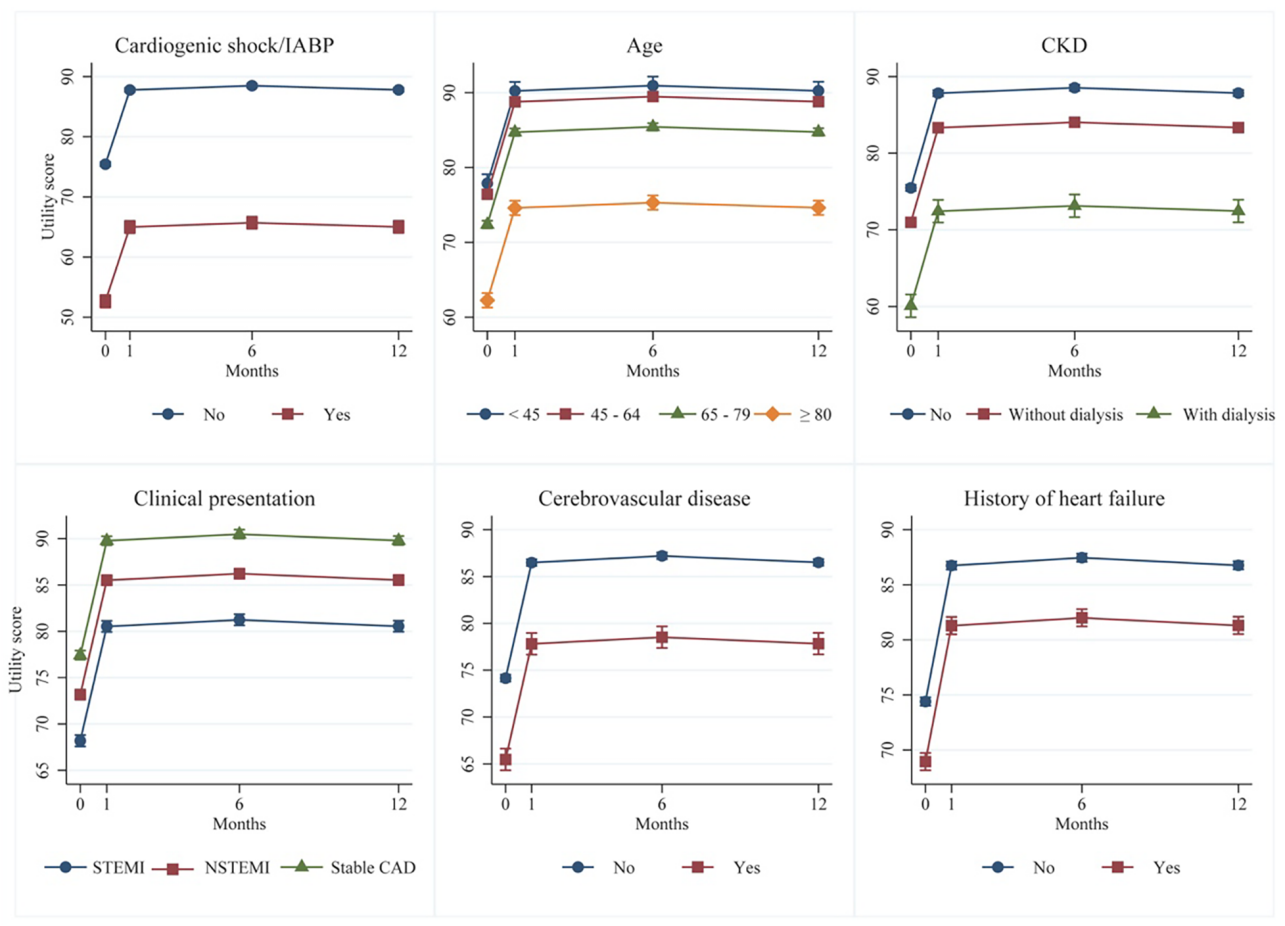
Six clinical risk factors associated with less improvement of HRQoLs after PCI: a multivariate mixed-effect regression.

Of the 16 factors noted above, only five were shown in the multivariate analysis to be significantly associated with HRQoL improvement. These factors were: male vs. female, overweight status (BMI ≥23 vs. <23 kg/m^2^), dyslipidemia vs. non-dyslipidemia, radial vs. other accesses and angiographic success vs. failure, with coefficients (95% CI) of 3.4 (2.8, 4.0), 1.4 (0.8, 2.0), 1.9 (1.3, 2.5), 2.9 (2.3, 3.4) and 6.3 (5.1, 7.6), respectively. Conversely, the remaining eleven factors were negatively associated with HRQoL, with six first-rank factors being cardiogenic vs. non-cardiogenic shock/IABP support, old age-groups vs. <45 group, CKD vs. non-CKD, clinical presentation with STEMI, NSTEMI/UA vs. stable CAD, prior cerebrovascular vs. non-cerebrovascular disease and history vs. no-history of heart failure (see Figure 2). Patients with cardiogenic shock/IABP support had significantly lower HRQoLs, with coefficients (95% CI) of −22.8 (−23.8, −21.8) compared to patients without shock/IABP support. Patients aged ≥80, 65–79 and 45–64 years also had significantly lower HRQoLs, with coefficients (95% CI) of −15.6 (−17.2, −14.1), −5.5 (−6.8, −4.2) and −1.5 (−2.7, −0.2), respectively, when compared to patients younger than 45 years. Patients with STEMI and NSTEMI/UA presentation had significantly lower HRQoL with coefficients (95% CI) of −9.2 (−10.0, −8.5) and −4.3 (−4.9, −3.6), respectively, compared to patients with stable CAD. Patients with comorbidities such as CKD with or without dialysis, prior cerebrovascular disease, history of heart failure, DM, hypertension, and previous CABG also had significantly lower HRQoLs compared to patients without comorbidities. The coefficients (95% CI) for these factors were −15.4 (−16.9, −13.9), −4.5 (−5.1, −3.9), −8.7 (−9.9, −7.5), −5.5 (−6.3, −4.7), −2.1 (−2.7, −1.5), −1.0 (−1.6, −0.3) and −3.0 (−5.2, −0.9), respectively. Additionally, patients who experienced any complications during the PCI procedure had a significantly lower HRQoL coefficient (95% CI) of −2.1 (−3.3, −0.9) in comparison to patients without complications.

In terms of health insurance, there were no significant differences in HRQoL among individuals covered by universal coverage, government service/state enterprise or social security service. However, individuals who were uninsured or had to self-pay exhibited a significantly lower HRQoL coefficient (95% CI) of −2.4 (−4.0, −0.8) compared to those with universal coverage.

## Discussion

Analysis of this large nationwide registry provided compelling evidence of a significant and sustained improvement in patient quality of life following PCI which extended from discharge through at least the following 12 months. However, older patients and those with comorbidities experienced improvements in HRQoL which were less than those without these profiles.

The positive impact of PCI on HRQoL has been extensively studied and has influenced guidelines governing its performance (19, 23–25), recognising improved HRQoL as a primary treatment goal and benefit (12). However, the identification of factors that influence changes in HRQoL after PCI has remained limited in Asian countries. In this study, we found that male gender, being overweight, having dyslipidemia, utilising radial access and, importantly, angiographic success were all associated with improved HRQoL. The latter increased HRQoL score by about 10 points, which is a significant clinical improvement compared to angiographic failure. Generally, a change in HRQoL of 5 points is considered to be of minimal clinical significance (26, 27). It is worth noting that the impact of the identified factors remained consistent irrespective of CAD severity, procedural details, and type of health insurance.

This study revealed that male gender was associated with better HRQoL outcomes, even after adjusting for other clinical factors. This finding was consistent with previous reports showing that males experience greater benefit in terms of HRQoL after undergoing PCI, with decreased frequency of angina, increased physical functioning, and improved HRQoL compared to females (19, 28, 29). There are several reasons why males may have better outcomes than females after PCI. Males tend to develop CAD at an earlier age, resulting in the presence of fewer comorbidities and risk factors (30–32). They also generally have larger coronary arteries and less diffuse disease compared to females (30). Females tend to present with CAD at a later stage, resulting in delayed diagnosis and treatment (33). Additionally, males may have better access to healthcare, more favorable socioeconomic status, and different behavioural patterns, which can impact the outcomes of PCI (28, 34). It is important to note that the relationship between gender and outcome after PCI for CAD is complex and multifactorial. Further research is needed to better understand the differences and tailor treatments to optimise outcomes for all patients, regardless of gender.

In terms of the influence of age on HRQoL, we found that while older patients experienced improved HRQoL outcomes after PCI, that improvement was less than that seen in younger patients. This may be attributed to a higher prevalence of comorbidities, reduced baseline physical function and/or an increased risk of complications after PCI, all of which can impact HRQoL. However, this study's findings were consistent with previous studies that demonstrated improved HRQoL in the elderly after PCI (35, 36). Successful PCI can result in meaningful improvements in HRQoL even in octogenarians with comorbidities (37–39). However, it is important to consider individual patient characteristics and factors such as comorbidities, functional status, and frailty when evaluating the impact of PCI on HRQoL in octogenarians. It is noteworthy that these patients may recover physically more slowly, but still experience improved HRQoL with optimal medical therapy, healthcare professionals, and longer-term follow-up. Yan BP et al. (36), reported sustained improvement in HRQoL after PCI in elderly patients, comparable to that of younger patients. The authors suggest that age alone should not discourage revascularisation given its potential long-term benefits in HRQoL. Further studies are warranted to further improve selection criteria for the use of invasive revascularisation in the elderly.

Overweight and obesity (BMI > 23 kg/m^2^) are growing public health challenges, as they are closely associated with cardiovascular events mediated through risk factors such as DM, hypertension, and CKD (40). Several studies have investigated the impact of body weight on change in HRQoL associated with PCI (41–43). Previous research has shown that overweight and obese patients often exhibit better physical functioning and overall quality of life scores compared to those with normal or lower BMI (41, 43–45). Our study found that overweight patients experienced improved HRQoL after undergoing PCI. It is important to emphasise that the relationship between body weight and HRQoL outcome after PCI is complex and may be influenced by such factors as age, comorbidities, and socioeconomic status. But given the multiple negative impacts of overweight or obesity, we continue to encourage weight control to minimise risk factors associated with atherosclerotic CAD, aiming to prevent MACEs and premature deaths.

The relationship between dyslipidemia and HRQoL after PCI is not fully understood. However, the results of this study indicated that patients with dyslipidemia may benefit disproportionately in terms of HRQoL after undergoing PCI. This could be attributed to the fact that patients likely received statin therapy which also improves HRQoL by reducing angina frequency and enhancing physical functioning. Previous meta-analyses support their efficacy and recommend routine use of high-dose statin pretreatment in patients undergoing PCI regardless of clinical presentation (46–48). It is crucial for patients with dyslipidemia to receive optimal medical therapy (including lipid-lowering therapy) to mitigate cardiovascular risk, recurrent cardiovascular events, need for repeat revascularisation and improve overall health outcomes.

Clinical presentation plays a crucial role in determining HRQoL after PCI. Patients with more severe anginal symptoms at baseline may experience greater improvement in HRQoL after a successful PCI, as relief from angina can significantly impact their activity level and well-being (11). However, several studies show that patients with ACS, such as STEMI or NSTEMI/UA, have improvement in HRQoL but less than those with stable angina after PCI (25, 49). Similarly, in this study, patients with STEMI or NSTEMI/UA had lower HRQoL scores upon admission than others, and while improving after PCI, this was not as much as those of patients with stable angina. This could be due to the fact that these patients often have more severe CAD, higher risk profiles and undergo more complex procedures. In contrast, patients with stable CAD consistently experience decreased angina and improved HRQoL after PCI, although the benefits may be small (48). Importantly, though HRQoL improved after PCI, the effect did not last long. Healthcare professionals are needed to maintain and further enhance the HRQoL of these patients and consider introducing interventions immediately post-PCI (50).

Regarding coronary angiographic severity and procedural details, the only variables that had significant impact on HRQoL were radial access and angiographic success. Notably, almost half of this study's patients underwent PCI using radial access. It is associated with reduced bleeding complications, shorter hospital stays, and improved patient comfort compared to femoral access, and so positively impact HRQoL outcomes. However, the choice of access site depends on multiple factors, including operator expertise, patient characteristics, and procedural complexity. Interestingly, the number of vessels treated, number of stents used, severity of lesions, additional adjunctive plaque modification devices, and treatment CTO lesions were not associated with changes in HRQoL after PCI. As long as patients had angiographic success with PCI, they had improved HRQoL outcomes based on relief of symptoms and enhancement of overall cardiac function. Avoidance of complications during PCI, particularly cardiogenic shock requiring IABP support, is crucial as these can impact HRQoL, lead to longer hospital stays, increase healthcare utilisation, and adversely affect cardiac function, all of which can negatively impact HRQoL. Prompt recognition and management of complications during PCI are important to minimise their impact on HRQoL outcomes.

The influence of type of health insurance on HRQoL in patients with CAD after PCI is complex and varies across countries. While expanding insurance coverage is crucial for improving access to care, efforts should also focus on enhancing the quality of primary health care. Our result suggests that universal coverage, government service/state enterprise, or social security service were associated with similar effects on improving HRQoL outcomes after PCI, while those uninsured or self-paying tended to have poorer baseline and post-PCI HRQoL outcomes. It is important to note that access to appropriate medical care and follow-up after PCI, regardless of insurance status, plays a critical role in determining quality of life in patients with CAD.

### Study limitations

While studies utilising PCI registries can provide valuable insights into the relationship between PCI and changes in HRQoL of patients with CAD, there are several limitations that need to be considered when interpreting the results. Firstly, this study was conducted in a Thai population and hospital system, and it is possible that unique characteristics of the patients, physicians, or hospitals may limit the generalisability of these findings. Secondly, not all patients in whom PCI was indicated chose to have the procedure. This may have introduced a selection bias among those who voluntarily opted for PCI. Thirdly, there is a lack of standardised measures for HRQoL or utility scores, as multiple different measures are available. In this study, we used EQ-5D-5l and EQ-VAS which limits comparability with the results of other studies. Fourthly, this study may not have fully accounted for all variables that could potentially confound HRQoL outcomes, such as socioeconomic status, psychosocial status, or concomitant treatments including risk factor control. Socioeconomic status, including factors such as income, education level, and social support, can also impact HRQoL. Patients facing challenges in accessing healthcare services, adhering to medications, and maintaining healthy lifestyle behaviours may experience adverse effects on HRQoL. It is essential for physicians to advise patients to actively engage in risk factor management after PCI to mitigate disease progression and prevent future cardiovascular events. Lastly, follow-up was incomplete, with 1.2% of subjects not completing the 12-month assessment despite the study team's best efforts to contact each patient by phone. Though a small percentage, this incomplete follow-up may have introduced bias to the findings. Further research, using rigorous study designs and standardised measures, is needed to better understand the impact of PCI on changes in HRQoL in patients with CAD. By addressing these limitations, future studies can provide more comprehensive insights into associations between PCI and HRQoL outcomes, and so help guide clinical practice and improve patient care.

## Conclusion

This analysis of subjects from a nationwide PCI registry in Thailand provides additional evidence of the beneficial impact of PCI on the quality of life of patients with CAD, irrespective of clinical presentation, as observed over a 1-year follow-up period. This study identified several independent factors associated with PCI-related improvements in HRQoL, including angiographic success, male gender, overweight status, dyslipidemia, and radial access. Awareness of these associations may assist clinicians in identifying patients likely to have better or worse HRQoL outcomes after PCI, and in the tailoring of interventions to optimise HRQoL.

## Data Availability

The raw data supporting the conclusions of this article will be made available by the authors, without undue reservation.
