# Supplementary material for: Factors associated with health-related quality of life in patients undergoing percutaneous coronary intervention: Thai PCI registry
Source: Front Cardiovasc Med. 2023 Nov 8;10:1260993. doi: 10.3389/fcvm.2023.1260993 (PMC10663305; doi:10.3389/fcvm.2023.1260993)
Supplement: Supplementary file 1 [file Datasheet1.docx]

**Supplementary Table 1.** HRQoL measured by EQ-5D-5L and EQ-5D-VAS at admission, discharge, 6 and 12 months follow-up.

| HRQoL | Admission | Discharge | Follow-up | Follow-up |
| --- | --- | --- | --- | --- |
|  |  |  | 6 months | 12 months |
| *EQ-5D summary score* |  |  |  |  |
| Visual analogue | 66.6 ± 19.6 | 81.9 ± 13.8 | 86.1 ± 12.3 | 88.0 ± 11.7 |
| *EQ-5D individual components* |  |  |  |  |
| **Mobility, n (%)** |  |  |  |  |
| I have no problems in walking about | 9,488 (52.6) | 11,708 (61.8) | 13,567 (74.8) | 13,797 (77.8) |
| I have slight problems in walking about | 4,094 (22.7) | 5,336 (28.2) | 3,518 (19.4) | 3,050 (17.2) |
| I have moderate problems in walking about | 2,733 (15.1) | 1,285 (6.8) | 715 (3.9) | 597 (3.4) |
| I have severe problems in walking about | 1,233 (6.8) | 365 (1.9) | 227 (1.3) | 198 (1.1) |
| I am unable to walk about | 492 (2.7) | 237 (1.3) | 114 (0.6) | 97 (0.5) |
| **Self-Care, n (%)** |  |  |  |  |
| I have no problems washing or dressing myself | 10,137 (56.2) | 12,304 (65.0) | 13,957 (76.9) | 14,107 (79.5) |
| I have slight problems washing or dressing myself | 4,210 (23.3) | 5,228 (27.6) | 3,425 (18.9) | 2,955 (16.7) |
| I have moderate problems washing or dressing myself | 2,313 (12.8) | 987 (5.2) | 518 (2.9) | 493 (2.8) |
| I have severe problems washing or dressing myself | 941 (5.2) | 223 (1.2) | 160 (0.9) | 114 (0.6) |
| I am unable to wash or dress myself | 439 (2.4) | 189 (1.0) | 81 (0.4) | 70 (0.4) |
| **Usual Activities, n (%)** |  |  |  |  |
| I have no problems doing my usual activities | 8,261 (45.8) | 10,866 (57.4) | 13,009 (71.7) | 13,357 (75.3) |
| I have slight problems doing my usual activities | 4,685 (26.0) | 6,013 (31.8) | 4,049 (22.3) | 3,552 (20.0) |
| I have moderate problems doing my usual activities | 2,988 (16.6) | 1,461 (7.7) | 758 (4.2) | 591 (3.3) |
| I have severe problems washing or dressing myself | 1,453 (8.1) | 391 (2.1) | 214 (1.2) | 156 (0.9) |
| I am unable to wash or dress myself | 653 (3.6) | 200 (1.1) | 111 (0.6) | 83 (0.5) |
| **Pain/Discomfort, n (%)** |  |  |  |  |
| I have no pain or discomfort | 6,527 (36.2) | 10,922 (57.7) | 13,019 (71.8) | 13,507 (76.1) |
| I have slight pain or discomfort | 5,529 (30.6) | 6,624 (35.0) | 4,358 (24.0) | 3,662 (20.6) |
| I have moderate pain or discomfort | 3,393 (18.8) | 1,117 (5.9) | 610 (3.4) | 453 (2.6) |
| I have severe pain or discomfort | 1,880 (10.4) | 197 (1.0) | 121 (0.7) | 96 (0.5) |
| I have extreme pain or discomfort | 711 (3.9) | 71 (0.4) | 33 (0.2) | 21 (0.1) |
| **Anxiety/Depression, n (%)** |  |  |  |  |
| I am not anxious or depressed | 7,475 (41.4) | 11,781 (62.2) | 14,451 (79.7) | 14,673 (82.7) |
| I am slightly anxious or depressed | 5,702 (31.6) | 6,046 (31.9) | 3,221 (17.8) | 2,697 (15.2) |
| I am moderately anxious or depressed | 2,982 (16.5) | 891 (4.7) | 358 (2.0) | 296 (1.7) |
| I am severely anxious or depressed | 1,425 (7.9) | 150 (0.8) | 80 (0.4) | 53 (0.3) |
| I am extremely anxious or depressed | 456 (2.5) | 63 (0.3) | 31 (0.2) | 20 (0.1) |

HRQoL, Health-related quality of life; EQ-5D-5L, EuroQoL-5 Dimensions 5-Level; EQ-5D-VAS, EuroQoL Visual Analogue Scale.
